# Exploring community-based reporting of livestock abortions for rift valley fever and brucellosis surveillance in Uganda: a pilot study

**DOI:** 10.1038/s41598-025-26710-w

**Published:** 2025-11-28

**Authors:** Abel W. Walekhwa, Andrew J. K. Conlan, Stella Acaye Atim, Anna Rose Ademun, Emmanuel Hasahya, James L. N. Wood, Lawrence Mugisha

**Affiliations:** 1 Disease Dynamics Unit, Department of Veterinary Medicine, University of Cambridge, Cambridge, UK; 2 IDEMU Mathematical Modelling Unit, Department of Epidemiology and Biostatistics, Makerere University School of Public Health, P.O. Box 7072, Kampala, Uganda; 3 International Livestock Research Institute (ILRI), Kampala, Uganda; 4 Makerere University College of Veterinary Medicine, Animal Resources and Biosecurity, Kampala, Uganda; 5 Ecohealth Research Group, Conservation and Ecosystem Health Alliance (CEHA), Kampala, Uganda; 6 National Animal Disease Diagnostics and Epidemiology Centre, Ministry of Agriculture, Animal Industry and Fisheries, Entebbe, Uganda; 7 MRC/UVRI and LSHTM Uganda Research Unit, Entebbe, Uganda

**Keywords:** Livestock abortions, Rift valley fever, Brucellosis, Isingiro district, Uganda, Molecular biology, Diseases

## Abstract

Rift Valley fever (RVF) and brucellosis are serious zoonotic diseases with significant public health and economic consequences. In livestock, both diseases are associated with abortions that are not routinely reported as part of disease surveillance. We piloted a community-driven call centre initiative in Isingiro District, Uganda, from March to June 2023, aimed at facilitating the reporting of livestock abortions. The community call centre was promoted through stakeholder engagements, social media campaigns, and targeted sensitisation materials.

Over three months, we received 53 alerts reporting 423 livestock abortions. We investigated 78% of these alerts and collected 200 serum samples. Of these, 184 samples were tested via ELISA for RVF virus antibodies (IgG and IgM), while all 200 were screened for anti-Brucella (IgG) antibodies using validated commercial kits. The proportion of aborting livestock testing positive for IgG antibodies to Rift Valley fever virus (RVFV) was 38% [95% CI 29–47] in cattle, 33% [95% CI 14–61] in sheep, and 20% [95% CI 12–31] in goats. For IgM, sheep showed the highest proportion at 8% [95% CI 1–35], followed by cattle at 2% [95% CI 1–6]. Further analysis showed that cattle had a 2.9-fold elevated odds of RVF IgG seropositivity relative to sheep and goats (OR = 2.9, 95% CI 1.27–7.07, *P* = 0.014). The proportion of aborting livestock testing positive for IgG antibodies to brucellosis was 36% [95% CI 25–49] in goats and 16% [95% CI 11–23] in cattle, with no evidence of antibodies found in the sampled sheep.

Our pilot study revealed high seropositivity to RVFV and brucellosis in cattle, sheep, and goats that had recently experienced abortions. This study demonstrates that early reporting of abortions by communities, followed by immediate collection of samples for diagnosis, could facilitate early detection and response to outbreaks. Furthermore, the response registered at the call centre by livestock owners has the potential for collaborative efforts to establish disease reporting and surveillance, thereby enabling more timely interventions.

**Supplementary Information:**

The online version contains supplementary material available at 10.1038/s41598-025-26710-w.

## Introduction

Although the World Health Organisation (WHO) recommends that countries and institutions should have early warning mechanisms to track zoonotic diseases, including Rift Valley fever (RVF) and brucellosis, many outbreaks are detected late^1^. The issue of timeliness is emphasised in the 7-1-7 framework, which states that countries should detect disease outbreaks within seven days, then notify different stakeholders within one day, and finally institute public health interventions within a further seven days^1^. However, in Uganda, the surveillance system remains passive^2^. This languid pace is more pronounced in the animal health sector, with long detection durations; one study reported a detection duration of 52 days^3^ and another of 83 days^2^ for anthrax, as opposed to the recommended seven days. For RVF in particular, previous evaluations of the 7–1-7 framework for Uganda have estimated detection delays of up to 43 days^4^. In this process, smallholder farmers lose livestock, which are the most reliable source of livelihood for food, education, and cultural obligations^5^. Such livestock losses lead to economic disruptions that complicate already constrained, low-resource settings and households^6,7^. This calls for urgent, community-centric, affordable approaches to support early detection of livestock diseases, which were a primary motivation for this exploratory pilot study^8^.

The absence of early warning mechanisms for some zoonoses (such as RVF and brucellosis) is primarily attributed to the lack of resources (particularly in low- and middle-income countries) to carry out systematic surveillance of RVF in known animal hosts. This presents a significant barrier to the control of RVF and brucellosis^9^.

Abortions in livestock are one of the most significant sources of economic impact^10–12^ and the most visible clinical signs of infection with both RVF and brucellosis. Abortions, defined as the loss of a foetus between day 42 and 271 of pregnancy^13^, can also be caused by several other factors, including genetic defects, injury, and poor nutrition^14–16^. In contrast to many of these factors, abortions caused by infectious diseases would be expected to cluster in space and time^10^. Thus, an anomalous increase in livestock abortions could provide an early warning signal of the circulation of infectious diseases within livestock and human communities^17^.

There is a growing body of scientific knowledge regarding livestock abortions, given their economic and production implications. For example, in northern Tanzania, over ten key pathogens (including RVFV and *Brucella spp*) were detected among livestock through abortion surveillance^16^. A related study documented an annual economic net loss of about USD 263 million to the government of Tanzania due to livestock abortions^5^. However, in Uganda, where brucellosis is declared an endemic disease^18–20^ and RVF is thought to be silently circulating, there has been minimal attention paid to tracking livestock abortions^21^. Livestock abortions are neither routinely documented nor reported for action, yet they could be leveraged as cost-effective proxies for arbovirus surveillance in ruminants^22^. In this project, we piloted a community-led reporting system for abortions. The objective of this study was to estimate the proportion of animals that have recently experienced abortions that are seropositive for RVF and brucellosis.

Thumbi et al. piloted a similar syndromic surveillance system in rural areas of Kenya that showed that animal health illness events were 15 times more likely to be reported by phone-based surveillance than by home visits by veterinary health workers^23^. We built on the methodology of Thumbi et al., adding publicity through social media channels, community sensitisation to increase awareness, and laboratory confirmation of reported and investigated abortion cases for RVF and Brucella. RVF and brucellosis have been reported to be the two leading causes of livestock abortions identified through abortion surveillance in neighbouring Tanzania^24^. RVF is a zoonotic viral infection caused by the Rift Valley fever virus (RVFV), a member of the genus *Phlebovirus* and family *Bunyaviridae,* which is transmitted by mosquitoes^25,26^.

The zoonotic risk posed by RVFV and the uncertainty about the extent of its circulation in Uganda demonstrate the need for a more systematic method of surveillance, and this was the primary motivating factor for this study. Brucellosis is a bacterial disease caused by various *Brucella* species and mainly affects cattle, swine, goats, sheep, and dogs (WHO, 2020). In contrast to RVF, Brucella is well established to have a high prevalence in Uganda^27^ compared to other infections associated with abortion storms^28–30^. Given these factors and the evidence for the importance of both RVF and Brucella in neighbouring countries, we prioritised screening for these two infections to collect baseline data on the diagnostic status of animals recently experiencing abortions. We aimed to collect samples from at least one recently aborted animal of each species reported by the smallholder households.

## Results

### Call centre alerts

At the planning phase of the fieldwork (21 January 2023), there were a small number (10) of reports of livestock abortions that had been recorded by the DVO’s office independently of our study (Fig. 3). During the pilot phase, we received a total of 53 call alerts, leading to 423 abortions reported from 53 farm/herd owners. One hundred eighty-four (184) out of 423 abortions reported were investigated for this paper, and 184 samples were collected (Table 1). Of the 184 samples, 10 were collected from 10 abortions that occurred before the establishment of the call centre, from cattle reported from Bugango Town Council (6/10) and Mbaare Sub-county (4/10). Four and six of these ten samples were estimated to be from the early stage and middle of pregnancy, respectively (S1). All of the call alerts were made through telephone calls rather than social media platforms like Facebook and WhatsApp. There was strong interest in participating in this pilot and high demand from smallholder farmers to understand the cause of livestock abortions. This pilot had been planned to take place over six months, but due to the high rate of reports and delays in starting due to logistical factors, it was completed within three months (Fig. 2).

**Table 1:** Total number of abortion cases reported at the call centre.

Call centre alert #	Geographical locations (sub counties)	Number of reported abortions (N)	Abortion cases investigated/ Sera samples collected (n)	Proportion sampled (%)
1	Masha	4	*	
3	Rwentango	10	*	
4	Ngarama	21	13	57
5	Ngarama	2		
6	Kashumba	4	40	66
7	Kashumba	3		
8	Kashumba	1		
9	Kashumba	30		
10	Kashumba	3		
11	Kashumba	5		
12	Kashumba	4		
13	Kashumba	8		
14	Kashumba	3		
15	Bugango	2	*	
16	Bugango T/C	6	14	100
17	Bugango T/C	3		
50	Bugango T/C	5		
18	Mbaare	10	14	78
19	Mbaare	8		
20	Nakivale	1	*	
21	Nakivale	2	*	
22	Nakivale	1	*	
23	Nakivale	2	*	
24	Kakamba	24	17	71
25	Rushasha	28	12	43
26	Endizi T/C	10	1	2
27	Endizi T/C	8		
28	Endizi T/C	6		
29	Endizi T/C	5		
30	Endizi T/C	6		
31	Endizi T/C	5		
32	Endizi T/C	3		
35	Endizi T/C	5		
36	Endizi T/C	14		
37	Endizi T/C	5		
38	Endizi T/C	4		
39	Endizi T/C	3		
40	Endizi subcounty	16	1	3
41	Endizi subcounty	3		
42	Endizi subcounty	8		
43	Endizi subcounty	4		
44	Endizi subcounty	3		
45	Endizi subcounty	6		
46	Rugaga	9	43	72
47	Rugaga	25		
48	Rugaga	23		
49	Rugaga	3		
51	Ruborogota	22	19	86
52	Kikagati	30	6	19
53	Kikagati	2		
33	Rwanjogyera	4	4	80
34	Rwanjogyera	1		
	Total	423	184	

*Lost samples due to labelling loss during transportation to the national laboratory.

Regarding the delay between the date of reporting/alert and investigation, the average delay was 9.2 days (SD ± 34.6). However, this figure is inflated due to the inclusion of reports submitted before the call centre’s establishment. After establishing the call centre, the majority of the alerts (78%) were investigated within less than 24 h (Fig. 4).

### Demographic data for livestock

Of the abortion samples collected, the majority, 106/184 (58%), originated from cattle, followed by goats at 66/184 (36%). Among the cattle, for which breed data were available (n = 52), 25 (48%) were crossbred and 22 (42%) were local breeds. None of the smallholder farmers reported any history of RVF vaccination, and the majority (98%) indicated no history of cross-border or inter-district livestock movement.

At the farm level, the reported clinical signs among affected animals included sudden onset of abortion in pregnant animals, weakness, unsteady gait, mucopurulent nasal discharge, foul-smelling diarrhoea, and high fever. Observed environmental and climatic factors in the vicinity of livestock farms and homesteads included the presence of bushes, recent rainfall (within the previous 14 days), dense forest cover, and stagnant water (Table 1, Table 2, Fig. 2).

**Table 2:** Socio-demographic characteristics of respondents.

Variable	Frequency (n)	Percentages (%)
Animal species tested for RVF		
Cattle	106	58
Goats	66	36
Sheep	12	7
Animal breeds in the study area (n = 52)		
Exotic	5	10
Crossbreed/hybrid	25	48
Locals (Ankole (*Bos taurus indicus),* East African shorthorn Zebu (*Bos indicus*))	22	42
Gender of respondent		
Female	26	14
Male	158	86
Means of reporting to the call centre (n = 52)		
Phone Calls	52	100
Number of livestock abortions reported (n = 52)		
Median (4.0 IQR 3.0)		
Occupation of the respondent		
Transport cyclist	2	1
Teacher	6	3
Small scale farmer	5	3
Herdsmen	159	86
Political leader	5	3
Others (religious leader, soldier, business)	4	2
Education level of the respondent		
Advanced level	14	8
Bachelors	5	3
Certificate	13	7
Diploma	12	7
No education	81	44
Ordinary level	58	32
Masters degree	1	0
Household size		
Median; 10 IQR 4.25		
Animal Clinical presentations at farm level*		
W	95	52
X	29	16
Y	18	10
Z	24	13
I don’t know	18	10
Stages of the pregnancy		
Early stage ( 1–3 months)	73	40
Middle stage (4–6 months)	62	34
Late stage (7–9 months)	45	24
Not sure	4	2
Environmental Conditions around the farm**		
A	81	44
B	52	28
C	15	8
D	36	20
History of animal movement		
No	181	98
Yes	3	2
Reported Animal vaccination status		
No	184	100
Total	184	100

*W: Sudden onset of abortion among pregnant animals, Weakness /Unsteady gait, Mucopurulent nasal discharge, Profuse fetid diarrhoea, High fever. X: Any three of the above symptoms, Y: Any two of the above symptoms, Z: Any one of the above symptoms.

**A: Bushes, Recent rainfall (less than 14 days), Heavy forests, Stagnant water. B: Only 3 present; Bushes, Stagnant water, Heavy forests. C: Only 2 present; Bushes, Recent rainfall (less than 14 days). D: Only 1 present.

### Seropositivity of RVF and brucellosis

The proportion of aborting livestock testing positive for RVF IgG antibodies in cattle was 38% [95% CI 29–47], 33% in sheep [95% CI14–61], and 20% in goats [95% CI 12–31]. The IgM seropositivity was lower, with 8% in sheep [95% CI 1–35], followed by cattle at 2% [95% CI 1–6]. Regarding brucellosis, goats had a seropositivity for IgG antibodies of 36% [95% CI 25–49; 21/58]; whereas 16% of cattle were positive [95% CI 11– 23; 20/125], and no sheep tested positive (Table 3).

**Table 3:** Serological results for RVF and brucellosis.

	Host	Apparent seropositivity (%) (95% CI)	Negative	Positive	Total number of animals, n (%)
RVF IgG	Cattle	38 (29–47)	66	40	106
	Goats	20 (12–31)	53	13	66
	Sheep	33 (14–61)	8	4	12
	Sub-total		127	57	184
RVF IgM	Cattle	2 (1–6)	104	2	106
	Goats	0 (0–0.1)	66	0	66
	Sheep	8 (1–35)	11	1	12
	Sub-total		181	3	184
IgG brucellosis ELISA	Cattle	16 (11–23)	106	20	125
	Goats	36 (25–49)	37	21	58
	Sheep	0 (0–0.2)	16	0	16
	Sub-total		159	41	200
Both IgG (RVF and brucellosis)	Cattle	9 (5–15)	54	9	106
	Goats	2 (0–8)	42	1	66
	Sheep	25 (9–53)	5	3	12
	Sub-total		101	13	184

Serological analysis further revealed a notable pattern suggestive of possible sequential exposure to both RVFV and *Brucella spp.,* where residual antibodies from prior infections may persist. Evidence of exposure was higher in cattle, 9% (95% CI 5–15%; 9/106), compared to goats at 2% (95% CI 0–8%; 1/66) (Table 3).

### Risk factor analysis

Multivariate logistic regression analysis identified host species as a primary determinant of RVF infection in our study population, underscoring that cattle may be the most critical host population to target in surveillance and vaccination strategies. Cattle exhibited a 2.9-fold elevated odds of RVF IgG seropositivity relative to sheep and goats (OR = 2.9, 95% CI: 1.27–7.07, *P* = 0.014), with CI excluding unity and statistical significance affirmed at *p* < 0.05. In contrast, the presence of vector-favourable environmental conditions proximal to farms or herds, encompassing bushes, recent rainfall (< 14 days), dense forests, or stagnant water, had a lower odds ratio and was not statistically significant at the 95% level (OR = 1.5, 95% CI 0.6–4.0; *P* = 0.4). Similarly, a history of livestock movement was linked to a threefold increase in the odds ratio but was also not statistically significant (OR = 3.0, 95% CI 0.1–71.0; *P* = 0.5) (Tables 4 and 5).

**Table 4:** Univariate analysis for RVF model variables.

Predictor variable	Estimate	Std.error	Z-value	*p*.value
Host_rvf Goats: (Intercept)*	− 1.405	0.31	− 4.541	0
Host_rvf Cattle	0.905	0.369	2.453	0.014
host_rvf Sheep	0.712	0.686	1.038	0.299
Stage_pregnancyLate stage (1–3 months)*	− 0.537	0.414	− 1.297	0.195
(Intercept)	− 0.474	0.241	− 1.971	0.049
Stage_pregnancyLate stage (7–9 months)	− 0.537	0.414	− 1.297	0.195
Stage_pregnancyMiddle stage (4–6 months)	− 0.499	0.373	− 1.338	0.181
Stage_pregnancy Not sure	− 16.092	1199.772	− 0.013	0.989
Environmental_features A (Intercept)*	− 1.115	0.258	− 4.328	0
Environmental_features B	0.479	0.389	1.232	0.218
Environmental_features C	− 0.271	0.695	− 0.39	0.696
Environmental_features D	0.892	0.423	2.109	0.035
History_animal_movement No (Intercept)*	− 0.803	0.161	− 4.993	0
History_animal_movementYes	0.110	1.235	0.089	0.929
Number_rvf_vaccinated Yes (Intercept)	− 0.793	0.16	− 4.969	0
Number_rvf_vaccinated No	− 0.230	14.712	− 0.016	0.988
destination_animalmovementNo(Intercept)	23.566	79,462.019	0	1
destination_animalmovementIsingiro	− 47.132	112,376.255	0	1
destination_animalmovementTanzania	− 47.131	112,376.255	0	1
subcountyKabingoTC (Intercept)	1.28E− 15	0.535	0	1
subcountyEndinzi	− 16.566	2399.545	− 0.007	0.994
subcountyEndinzi Town council	− 16.566	2399.545	− 0.007	0.994
subcountyKakamba	− 1.540	0.831	− 1.854	0.064
subcountyKashumba	− 0.619	0.629	− 0.984	0.325
subcountyKikagati	− 16.566	979.61	− 0.017	0.987
subcountyMbaare	− 0.288	0.76	− 0.379	0.705
subcountyNgarama	− 1.704	0.936	− 1.821	0.069
subcountyRuborogota	− 0.318	0.708	− 0.45	0.653
subcountyRugaaga	− 0.949	0.633	− 1.498	0.134
subcountyRushasha	− 0.693	0.813	− 0.853	0.394
subcountyRwanjogyera	− 1.098	1.272	− 0.863	0.388

*Variables considered for final model building (*p* < 0.2).

**Table 5:** Multivariate regression mode outputfor IgG RVF occurrence and associated risk factors.

Variable	Number n	Estimate	Std Error	Z value	OR^*1*^	95% CI^*1*^	*p*-value
Animal hosts							
Goats	66	REF	–	–	–	–	–
Cattle	106	1.07	0.44	2.45	2.9	1.3,7.1	0.014*
Sheep	12	1.012	0.73	1.39	2.8	0.6, 11.3	0.2
Stage of animal pregnancy							
Early stage (1–3 months)	73	REF	–	–	–	–	–
Middle stage (4–6 months)	62	− 0.74	0.48	− 1.51	0.5	0.18, 1.2	0.13
Late stage (7-9 months)	45	− 0.56	0.41	− 1.37	0.6	0.3, 1.3	0.20
Not sure	4	− 16.77	1181.54	− 0.01	0.0	–	1.0
Environmental features at the farm/herd level							
A**	81	REF	–	–	–	–	–
B**	52	0.34	0.41	0.83	1.4	0.6, 3.2	0.41
C**	15	0.19	0.74	0.25	1.2	0.2, 4.8	0.80
D**	36	0.43	0.48	0.90	1.5	0.6, 4.0	0.37
SS							
No	181	REF	–	–	–	–	–
Yes	3	0.96	1.47	0.65	2.6	0.1, 71.0	0.52

^*1*^OR = Odds Ratio, CI = Confidence Interval, *Statistically significant variable. **A: Bushes, Recent rainfall (less than 14 days), Heavy forests, Stagnant water. B: Only 03 present; Bushes, Stagnant water, Heavy forests. C: Only two present; Bushes, Recent rainfall (less than 14 days). D: Only one present.

As a sensitivity analysis, we refitted the multivariate model, excluding the small number (10) of reports where the delay between alert and investigation was greater than 14 days. The qualitative results in terms of identified risk factors and magnitude of effects were unchanged in the smaller data set. For brucellosis, no variables passed the (generous) univariable screen, so we did not proceed to multivariate analysis (S2).

### Challenges faced in piloting the call centre

Several operational hurdles were associated with piloting the call centre. The most pressing issue was the absence of dedicated veterinary officers in certain sub-counties, coupled with instances where a single officer was responsible for multiple sub-counties, which substantially compromised the timeliness of response to alerts. The structural shortfall in human resources not only delayed field investigations into reported abortion cases but also heightened the risk of under-detection and unchecked transmission of abortigenic pathogens within study populations, thereby undermining the call centre’s role in facilitating rapid epidemiological intelligence gathering and early intervention. To address this gap, we recruited private veterinary officers/practitioners who sell veterinary services, including drugs, to smallholder farmers. The integration of private practitioners in surveillance could be adopted by the Ministry of Agriculture, Animal Industries, and Fisheries (MAAIF) to help meet the most pressing animal health needs of the community.

A second significant challenge was logistical constraints in sample transportation, exacerbated by the functional veterinary cold chain infrastructure, which was limited to only two sub-counties (Rugaga Town Council and Isingiro Town Council–Rweknkubo HCIV). These logistical issues impeded the reliable collection and preservation of sera and vaginal swabs for laboratory analysis. A more comprehensive evaluation of the capabilities, opportunities, and motivations for abortion surveillance is addressed in a forthcoming qualitative study (currently under review).

## Discussion

This pilot study adds to a growing body of evidence supporting the viability of community-led systems for reporting livestock abortions and the potential of such systems to detect evidence of infection with Rift Valley Fever (RVF) and other pathogens^16,20,24^. Following the establishment of a call centre in Isingiro District, abortion incidents were systematically reported and recorded. Serological screening revealed evidence of recent RVF infection (IgM positivity) in cattle and sheep but not in goats.

This pilot study contributes to a critical knowledge gap regarding screening recently aborted livestock. To the best of our knowledge, no such research has been done in Uganda.

Thomas et al.^16^ addressed the same basic question in northern Tanzania, a neighbouring region to our study area. Thomas et al.^16^ carried out both a cross-sectional survey of the general population and a cohort study recruiting animals that had recently experienced abortions over a period of three years. Thomas et al. were able to collect both acute (within 72 h of abortion) and convalescent sera (4–6 weeks later) to define a more rigorous attribution of infections to abortion events. However, they also report seroprevalence within acute samples (measured as positivity to IgG or IgM antibodies), which provides a point of comparison to our estimates for a sympatric population. Thomas et al. reported seroconversion to RVF in the aborted cattle cohort of 21.1% (95% CI 12–32), lower than the 40% (95% CI 30–49) of animals positive to IgG or IgM in our study. Seropositivity in goats and sheep is likewise higher in our study (20%; 95% CI (10–29) and 42% (95% CI 15–72) compared to 1.4% (95% CI 1–2) and 0% (95% CI 0–8), respectively. IgM positivity in goats and sheep, associated with more recent infection, was more consistent with our finding of only 1 seroconverted sheep (1/12) and no goats (0/66) with estimates of (0%; 95% CI 0–8%) in sheep and (2%; 95% CI 0.2–7) in goats from Thomas et al.

In contrast to the results from their abortion cohort study, Thomas et al. reported a far lower seropositivity of RVF in the general community reported estimates of 4.4% (95% CI 4–5), 1.4% (95% CI 1–2) and 2.6% (95% CI 2–3) in cattle, goats and sheep respectively. The higher proportions of RVF seropositivity seen in our study will likewise not be representative of prevalence within Uganda, given both the heterogeneity in risk of transmission (due to vector abundance) and likely higher risk of exposure in herds that have experienced recent abortions.

Nonetheless, the comparable patterns of seropositivity in cattle reported here, compared to those from a known endemic country (Tanzania), point to a potential hidden burden of infection in Uganda and the need for expanded surveillance in livestock.

As measured by seropositivity to IgG, this pilot study revealed that cattle that had experienced an abortion (within 14 days) exhibited a significantly elevated odds for seropositivity for RVF IgG antibodies relative to sheep and goats, suggestive of greater infection risk among cattle. This disparity may stem from inherent biological vulnerabilities in cattle, such as their propensity to sustain elevated viremia titers for a longer time, which may enhance transmission efficiency to vectors^31^. Behavioural factors, including foraging in mosquito-infested floodplains during pastoral movements, are likely to amplify their exposure to the Aedes and Culex species that propagate RVFV^32,33^. Comparable patterns of heightened seroprevalence in cattle have been documented across multiple African contexts, with estimates ranging from 18.8 to 42.9% in bovines, often surpassing those in ovine (2.7–28.0%) and caprine (2.4–9.3%) populations^34–36^. Of particular relevance is a multi-year serological survey in Uganda, encompassing districts akin to our study sites, which corroborated elevated RVF antibody detection in cattle^37^. Nevertheless, the finding that cattle are a more at-risk host for RVF diverges from the prevailing epidemiological consensus, which posits sheep and goats as more acutely vulnerable in mixed-herd settings, frequently manifesting severe clinical outcomes such as abortion storms and neonatal mortality, while cattle serve primarily as amplifiers with milder symptomatology^35,38–40^. This apparent incongruity may be attributable to the disproportionate representation of cattle in our sampled cohort, but also the possibility of recent recoveries from RVF infection as detected by IgG.

With respect to brucellosis, there is a paucity of studies focusing on animals that have recently experienced abortion. In our pilot study, goats exhibited a higher proportion of IgG-positive antibodies (36%, 95% CI 25–49) compared to cattle (16%, 95% CI 11–23). The study by Thomas et al.^16^ conducted in Northern Tanzania did not report IgG findings, precluding a direct comparison^16,17^. Nonetheless, Thomas et al.^16^ reported no seroconversion among goats and sheep but identified a 5.6% IgM seropositivity in cattle. As IgM was not assessed in our pilot study, these findings are not directly comparable. Given the limited evidence, the results of this pilot study underscore the need for further research focused on livestock that have recently aborted. Such investigations would help elucidate the burden of brucellosis and other causes of abortions during the active or recovery phase. Although previous studies have demonstrated the presence of brucellosis antibodies in the general population^41–43^, these estimates are not directly comparable to the subpopulation of animals experiencing abortions examined in our pilot study.

Through this pilot, we have demonstrated the feasibility (including measures of acceptability, stability, coverage, completeness of data, and linkage to testing services) of setting up a call centre for abortion reporting in Uganda. Such call centres are particularly valuable for animal disease surveillance in settings like Uganda, where the majority of livestock smallholder farmers have phones but do not necessarily have internet access^23^. When a similar pilot study was carried out in Kenya, the authors recommended pathogen-specific abortion reporting and investigation, which our work has contributed to^10^. Such call centres can provide added value to both smallholder farmers and policymakers. For example, smallholder farmers can seek technical guidance on farm management and have the opportunity to report any unusual or suspected conditions at the farm. At a policy level, they could complement existing surveillance infrastructure at Uganda’s Ministry of Health and the office of the Prime Minister, such as the national-level emergency operations centre^44^ and the National One Health Platform^45^, respectively. These organisations have technical staff who can analyse and interpret phone reports.

Building on the point of inter-ministrial/agency coordination above, the integration of livestock samples into Uganda’s existing Ministry of Health sample transport system represents an essential step toward operationalising a One Health approach^45^ for RVF and brucellosis. Using the National Laboratory Sample Transport System^46^, which already supports human disease diagnostics, enabled the timely and temperature-controlled movement of veterinary samples to the central reference laboratory. Similar integrated logistics models have enhanced zoonotic surveillance efficiency in Kenya and Tanzania^47,48^. This demonstrates the practicality of leveraging established human laboratory networks to support animal health surveillance, optimise resources, and strengthen cross-sectoral disease preparedness^49^.

Furthermore, from the animal health sector’s perspective, establishing a national-level abortion surveillance system could leverage existing systems, such as the electronic infectious diseases surveillance (eIDS) system under MAAIF. Setting up call centres has been previously championed under the Ministry of Health, Uganda during both peak moments of different epidemics, such as the SARS-CoV2 pandemic^50^, for Ebola Virus Disease^51^, and longer-term endemic infections, such as HIV/AIDS and Tuberculosis^52,53^. Looking beyond Uganda, neighbouring Kenya has already established such an integrated animal health system^54^. Establishing district-level call centres in DVO’s offices to facilitate livestock abortion reporting could provide a cost-effective and sustainable path to improve animal health surveillance in Uganda. This could be sustained through targeted abortion testing.

We found animals with both RVF and brucellosis antibodies. Given that our entry point was livestock abortions to confirm potential causes, our screening for RVF and brucellosis was necessary, as both are likely to contribute to the occurrence of abortions. This finding underscores the need to set up integrated livestock disease surveillance, which could be supported by the development and deployment of point-of-care diagnostics that have multiple assays. Early warning systems could be designed with different signals to distinguish the contribution of RVF to observed abortions in comparison to other abortion-causing pathogens. This has been recommended by other studies^55–57^.

From previous epidemiological studies, the stage of pregnancy and the geographic location (sub-counties) in which farms were located^58^ could have yielded valuable insights into the associations between abortion events and prior exposure to RVF or brucellosis infections. However, for our study, the stage of pregnancy did not achieve statistical significance, and subcounty variables were excluded from the final multivariate model at the stepwise selection procedure. These outcomes are likely attributable to the limited sample size (n = 184) in our study, necessitating cautious interpretation of these study results.

Our study had several strengths: Our pilot demonstrated that livestock abortion reporting through a call centre is operationally practical, acceptable to farmers and capable of generating timely surveillance data. We have also demonstrated that reporting abortion incidents by farmers to an established call centre could aid the diagnosis of brucellosis and RVF. Integrating animal sample transportation through the national human health sample transportation hub system was a novel approach that demonstrates the much-needed One Health implementation at the sub-national level. We have added a further justification for the prioritisation of phone-based reporting for community-level disease surveillance in low-income countries where internet/online platforms are not well developed. We developed RVF IEC materials and built capacity for public health and veterinary staff working with the Isingiro District, which is very important for the sustainability of livestock abortion surveillance.

However, there are some important limitations in our study; we conducted this study over a relatively short period (March–June 2023) and could therefore not measure any seasonal patterns in livestock abortions. Extending our study over (at least) a year and a wider geographic range could allow the association between abortion rates and RVF transmission to be modelled and the potential to train statistical models to identify early warning signals that do not depend on expensive diagnostics^55–57^. We were only able to screen for antibodies as evidence of infection (ELISA tests); the use of PCR/viral neutralisation tests could have allowed the confirmation of the presence of active infections in abortion cases. This study had a limited scope and budget to generate pilot data to inform the feasibility, design, and likely costs of a longer longitudinal study to test the association between changes in the rate of reporting of livestock abortions and microbiological confirmation of RVF infection. To distinguish between vaccine-elicited and naturally induced antibodies against RVF in this study population, we depended on farmers’ self-reported vaccination histories, with all participants denying any prior immunisation against RVF. This assertion is consistent with Uganda’s prevailing RVF control guidelines, which exclude vaccination as a recommended measure. Nonetheless, such a possibility cannot be entirely ruled out, given the porous and inadequately regulated borders shared with Tanzania, where RVF vaccination is actively practised. Our determination of natural infection is consistent with contemporaneous studies that employed RT-PCR to confirm recent or ongoing RVF infections in the same study area during analogous periods^37,59^. We only sampled animals with evidence of recent abortions, which denied us the opportunity to assess the risk of RVF in the general livestock population. Budget constraints were also the reason we could only screen for RVF and IgG Brucellosis (and left out IgM). At the same time, there are other pathogens (parasitic, fungal, viral, and bacterial) that are also known to cause abortions in livestock (in particular, bluetongue virus, Q fever, and *Campylobacter* spp.). Ticks are common in the study area, which could also contribute to abortions from other causes.

In conclusion, this study underscores the substantial willingness among smallholder farmers in Isingiro District, Uganda, to engage in voluntary reporting of livestock abortions, coupled with an apparent demand for diagnostic testing to elucidate underlying etiologies. Our findings reveal a pronounced association between recent abortions and RVF seropositivity in cattle, surpassing that observed in small ruminants (sheep and goats), thereby highlighting the potential efficacy of prioritising cattle surveillance in early detection for RVF circulation. Notably, the seropositivity estimates align closely with those reported among the general livestock population in endemic regions such as Kenya, suggesting latent RVF activity in Uganda despite its non-endemic classification. While this pilot investigation adhered to MAAIF guidelines by withholding immediate diagnostic feedback to participants, sustaining high participation rates in future programs will hinge on ensuring prompt follow-up investigations and transparent result dissemination. These insights not only advocate for integrated, farmer-centric surveillance frameworks to mitigate zoonotic threats but also pave the way for enhanced policy interventions in resource-limited settings, ultimately bolstering regional livestock health and human-animal disease prevention.

## Methods

### Study area

**Fig. 1:**
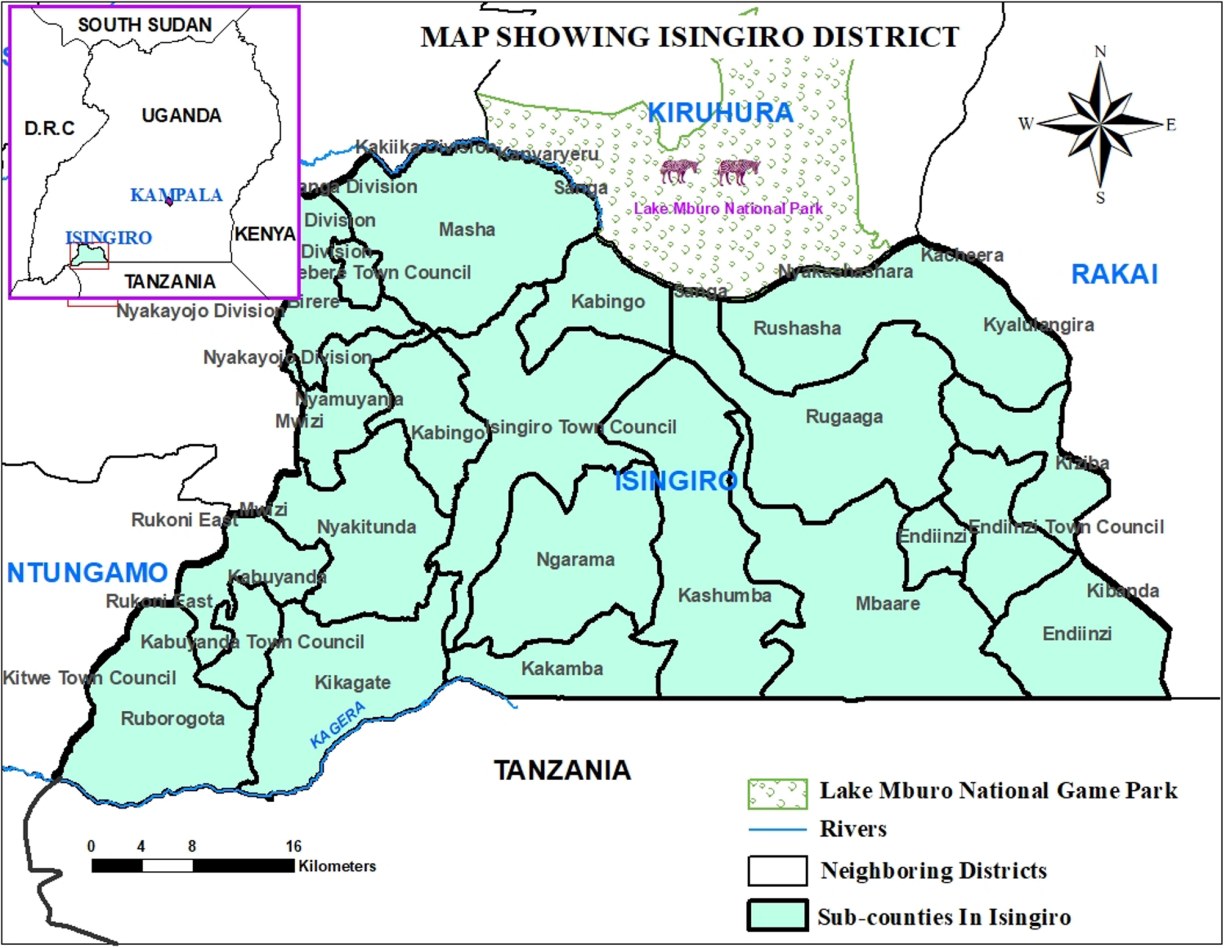
Map of Uganda showing Isingiro district and sites for sample collection.

Isingiro District (0.84° S, 30.80° E) is located in southwestern Uganda, about 297 kms from the capital city, Kampala, and 47 km from Mbarara city. Isingiro shares a border with Tanzania in the south and three further districts of Uganda: Kiruhura, Rakai, and Ntungamo (Fig. 1). These districts are characterised by livestock rearing being the major industry on which residents depend for their livelihood^60–62^. The district also borders Lake Mburo National Park, which harbours wildlife species susceptible to RVF. Both wildlife and livestock share common water sources along the shores of Lake Mburo and the Kagera River, which spans the district. Isingiro District has an estimated cattle, goats, and sheep population of 368,246, 422,108, and 88,621, respectively^63^, and a human population of 635,077 based on projections from the 2024 human census^58^. Isingiro District has thirty-five lower administrative units (sub-counties), with seventeen where animal rearing is conducted. The district experiences a tropical savannah climate with an average annual rainfall of 1200 mm, and a temperature range of 17–30 °C^64,65^. The district has two rainy seasons each year: March to April, and September to November. The people in this area rear livestock and practice crop agriculture as their main economic activities^66^.

This study was conducted among smallholder farmers (populations engaged in rearing livestock for their livelihoods). The population in our study area consists mainly of small-scale smallholder farmers who rear their livestock through a paddock system. Some of these smallholder farmers have established farms with access to water dams, and others operate free-range/tethering systems (where they move with their livestock from one area to another in search of pasture and water)^67,68^. In Isingiro, there are also educated (acquired formal education) smallholder farmers for whom farming is not their full-time job, who employ less educated people (casual workers) on their farms, who are directly engaged in day-to-day management and provide regular reports to the owners. The educated smallholder farmers are sometimes employed and based in urban settings like Kampala and Mbarara, and they manage their farms using mobile devices and online social media platforms like WhatsApp. Most of the smallholder farmers rear local and mixed breeds of cattle called Ankole-Friesian cross-breeds, which are preferred due to their resistance to ticks, drought, and varying weather conditions^68^. Some of these smallholder farmers engage in community-registered co-operative societies that enable them to access credit services and save their income from livestock.

### Study design

We conducted a cross-sectional study from March to June 2023.

### Sampling

Purposive sampling was employed to select the study location and participating subcounties (17 out of 33). Isingiro District was chosen as the study area owing to its history of RVF outbreaks in Uganda. For example, Ndishimye et al.^8^ documented that the district experienced the highest number of nationally reported RVF outbreaks between 2010 and 2024. Similarly, Arinaitwe et al^69^ identified RVF cases in Isingiro during their 2021 –2024 investigation, supported by additional evidence from historical data analyses^69^.

Sub-counties were selected based on elevated RVF risk factors, including high livestock density, proximity to Lake Mburo National Game Park, and the River Kagera, which serves as a water source for humans, livestock, and wildlife. The 17 chosen sub-counties are primarily focused on livestock rearing, in contrast to the 16 out of 33 sub-counties oriented toward crop production, which were excluded from the study.

On the farm, convenience sampling was utilised to collect at least one sample from each species, with priority given to livestock that most recently experienced abortion. All self-reported farms were investigated until the required sample size was attained. The target study population comprised recently aborted livestock, specifically cattle, goats, and sheep.

### Setting up a call centre–pilot

A call centre was established in consultation with livestock owners (smallholder farmers), local authorities, and District Veterinary Officers (DVO), to receive and record reported livestock abortions and other animal health conditions. The call centre was on average 12 kms from a typical farm in a sub-county. It would take, on average, 30–40 min for a veterinary officer using a motorcycle (the most common available method of transport) to reach the farthest farm in distant sub-counties like Kikagati, Rugaga Sub-County, or Endizi.

First, we conducted formal entry/information meetings to explain the study’s purpose, design, and approach, as well as the information to be collected, the expected participation, and the use of the call centre. Further meetings were then held with staff at the National Animal Diseases Diagnostics Epidemiological Centre (NADDEC), MAAIF, and local veterinary caregivers, briefing them on procedures for sample collection, packing, and transportation to the laboratory in Entebbe for analysis.

**Fig. 2:**
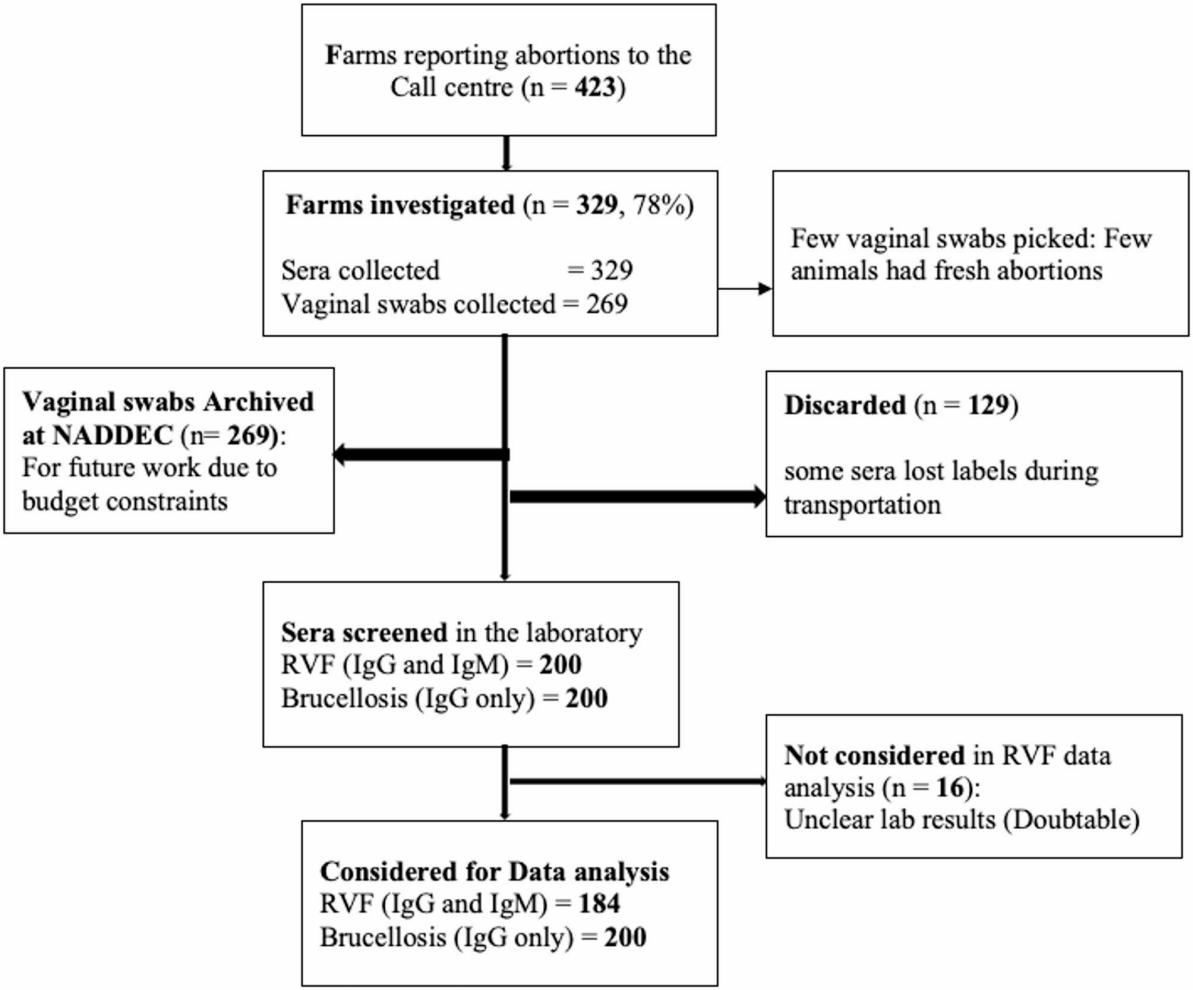
Flow chart of sample collection and analysis.

The call centre was officially established in the office of the DVO in the Isingiro District on 1st March 2023 and co-managed by the corresponding author and a local contact officer who served as a liaison person between this project and the DVO (Fig. 2). Following the official setup of the call centre at the district level, we were keen to follow up on abortion alerts that had occurred within the target 14-day period (14th February 2023 onwards). However, we also considered and responded to abortion cases that happened before the setup of the call centre.

Various stakeholder engagements, including meetings and mass sensitisation of livestock owners and local leaders, followed the establishment of the call centre. Information, Education, and Communication (IEC) materials on RVF and brucellosis were created. These materials were then distributed to public gatherings, barazas, and similar events across the study areas (sub-counties that rear livestock) to advocate for the reporting of abortions.

The contact officer was provided with a data collection tool, KoboCollect^70^, installed on Android phones, which enabled the tracking of all incoming calls regarding livestock abortions. During the design phase, we expected that these phones could facilitate reporting through phone calls, SMS, and social media alerts (WhatsApp, Facebook) in addition to verbal reporting through walk-ins to the office. We set up two days of orientation meetings with different stakeholders (Chief Administrative officer: in charge of technical management of the district, District Health Officer: in charge of the human well-being of the populations in the district, District Veterinary Officer: in charge of animal health in the district and District Chairperson: in charge of the political administration of the district) including the local research team that we had recruited to support in investigating/following up reported abortion cases. During these meetings, the participants were taken through the line-up of activities and their expected roles. This was necessary to ensure that stakeholders appreciated the scope of the project and would be more likely to support it. For the research team, this was necessary as it helped them to understand the project protocol. On the last day of the orientation, consent was sought, and a social media channel group (WhatsApp) was formed. This was done to coordinate the reporting of livestock abortion reports across the district. High-level coordination of activities and events was carried out through a separate social media group (WhatsApp group), which included the corresponding author, District Health Officer (DHO), DVO, Assistant District Health Officer-Environmental Health (ADHO-EH), call centre staff, and all research assistants. There were also weekly update meetings organised to review progress.

**Fig. 3:**
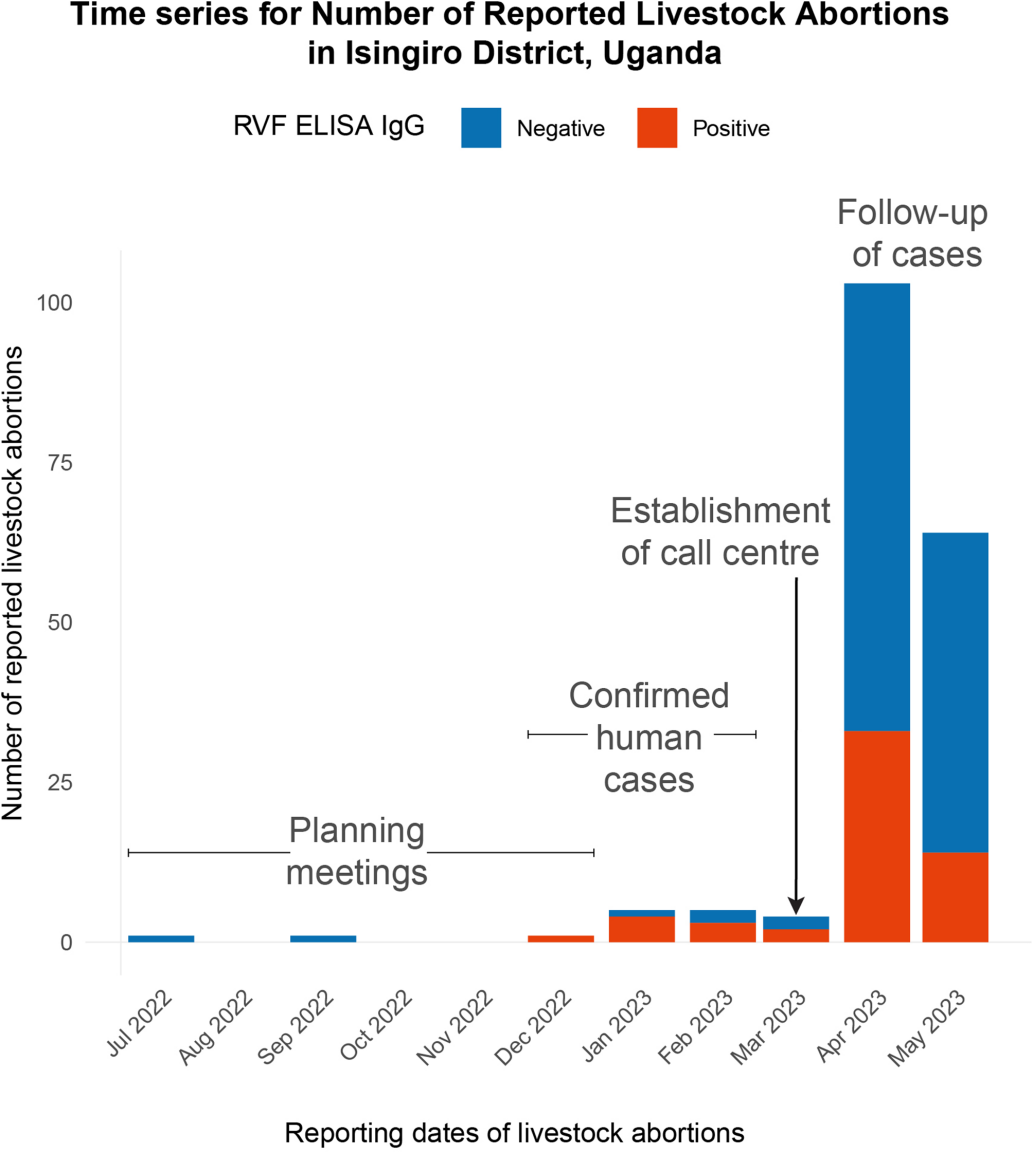
Time-series for livestock abortions reporting in Isingiro district, Uganda.

**Fig. 4:**
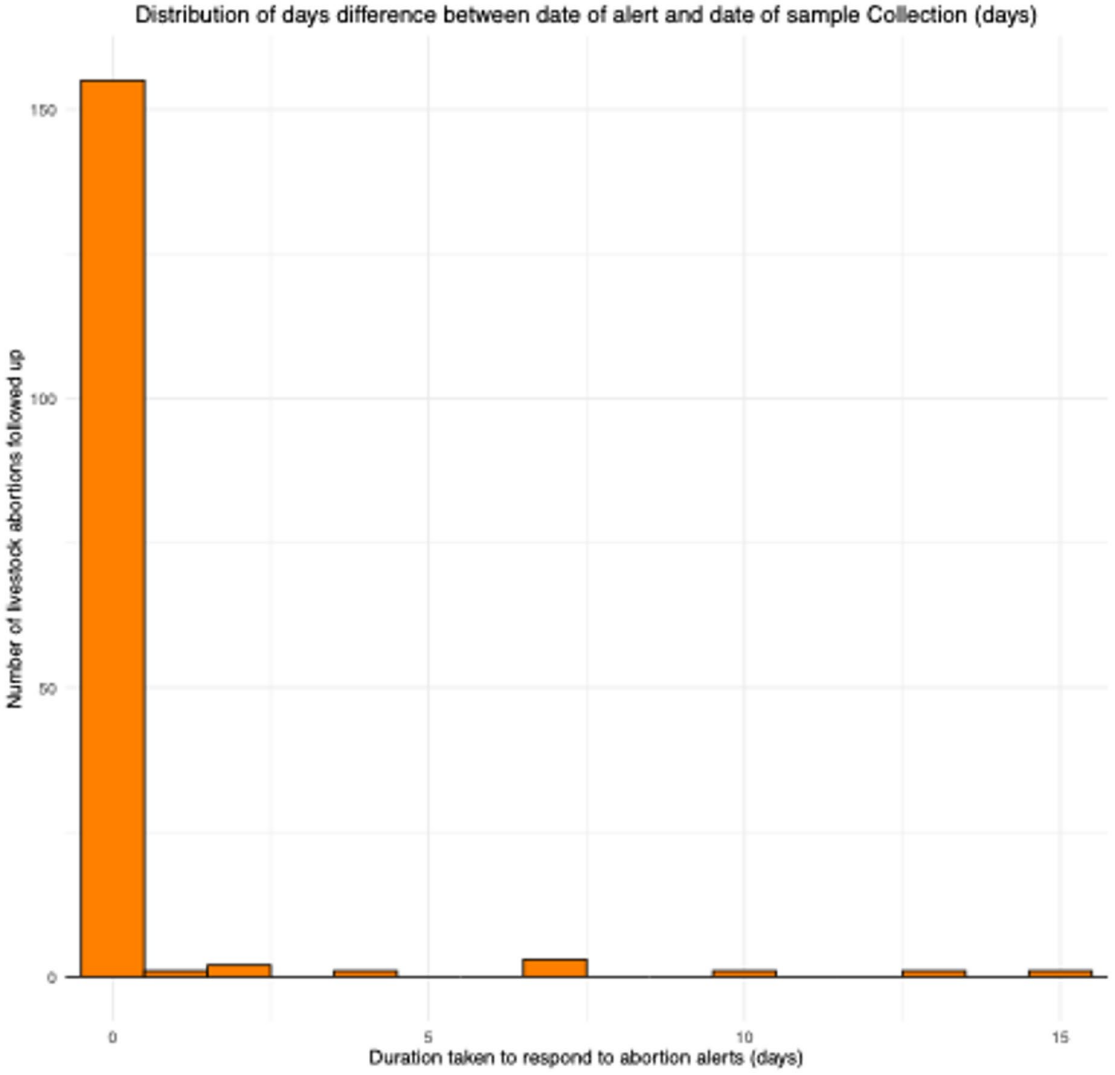
Delay between alert and investigation (days).

We provided two telephone numbers (the DVO and the contact officer) on the materials to enable livestock abortions to continue to be reported after the end of the project. IEC materials in the form of flyers and brochures (in English, Appendices: Fig. 3,4) were adapted from RVF risk communication materials created by the Ugandan Ministry of Health. These materials were distributed and displayed in public places in the community and provided to local authority leaders for use in addressing the public during community/social events like burials, ceremonies, radio talk shows, and community barazas. We also made announcements on local radio and carried out sensitisations with community health workers.

To determine the appropriate scale of the study, we conducted a sample size calculation to ensure we could estimate the expected proportion ($p$) in the area with a specified precision ($d=5\%$). We used the Kish-Leslie formula ($n = {z}^{2}pq/{d}^{2}$) where the desired confidence interval was taken to be 95% (5% significance level). The expected prevalence of RVF during abortions (effect size) was taken as 21% as estimated by de Glanville, Allan et al.^71^ in Tanzania (neighbouring country to Uganda). This calculation gave a target sample size of 325 for 80% statistical power.

### Field data collection

To provide capacity building in the local area and to build sustainability for our project, we identified and recruited veterinary and laboratory staff employed in the Isingiro District to join our project. Collaborating with staff who were already engaged in this study area was not only cost-effective but also proved beneficial, as it helped expedite activities. The local staff’s familiarity with the local geography facilitated the logistics of data collection. Through this project, we brought on board both veterinary staff working in public and private practice. In preparation for fieldwork, these veterinary and laboratory staff were given safety training on laboratory Standard Operating Procedures (SOPs), use of Personal Protective Equipment (PPE) and the handling and restraint of animals. These SOPs enabled staff to adhere to animal welfare principles while also ensuring personnel safety and adherence to biosecurity measures to curtail the spread of disease between farms. The team was also trained on the approved study protocol and data collection tools for two days.

The investigation team was provided with equipment and consumables for collecting sera and vaginal swabs from aborted livestock. We responded to 78% (329/423) of the reported alerts. This approach was intended to inspire smallholder farmers to report abortion alerts to the call centre. After every alert, there was an investigation by the corresponding author and the veterinary staff. During these investigations, a questionnaire was used to collect background information from the animal owners/smallholder farmers. The collected data included key demographic and epidemiological variables such as herd size, RVF vaccination history, alert channel used, environmental factors like stagnant water, presence of shrubs/forests, irrigation dams, vegetation cover, and economic activities. The blood and vaginal swab samples collected were transported in ice-conditioned icepacks (approximately four hours after sample collection) to Rwenkubo Health Centre IV (central laboratory hub for Isingiro District) and were stored at − 20 °C. This laboratory received daily vaginal swab samples from the veterinary staff. We collected 200 sera samples and 269 vaginal swabs and transported them to NADDEC for analysis, which were then tested for both RVF and Brucellosis. The transportation of samples was integrated into the already established Ministry of Health-accredited National Laboratory system (Hub)^46^ under cold chain conditions specified by the manufacturer to the central testing laboratory (NADDEC)^72^. Due to budget constraints, it was not possible to process these vaginal swabs; however, they have been stored for a future study should funds become available (Fig. 2).

### Laboratory analysis

Blood samples were tested by Enzyme-linked immunosorbent assay (ELISA) for anti-RVF and anti-Brucella antibodies using validated commercial kits. ELISA tests are World Organisation for Animal Health (WOAH) approved diagnostics for surveillance of RVF^73^ and, in Uganda, sufficient for confirmation of outbreaks as specified by WOAH^73^ and MAAIF protocols^74,75^. ELISA kits are commonly used in low-resource settings^76^ and are recommended by the World Organisation for Animal Health to estimate the prevalence of infection of RVF^77^. PCR testing or viral neutralisation could have proved virological confirmation of the presence of RVFV, but it was not possible in this study due to budget constraints.

For RVF, we used the RVF competitive multi-species ELISA Kit (ID VET, Montpellier, France)^77^. Although vaccination against RVF could stimulate the production of specific antibodies, producing a positive IgM test result^78^, the vaccine status of animals was verified, with smallholder farmers, with none reporting that they had purchased or used vaccines for RVF. *Brucella* assays were carried out using IBL-America IgG-ELISA Kits (Minneapolis, MN) as described by Nyamota et al.^79^.

## Statistical analysis

All data analyses were carried out using R statistical software version 4.3.1^80^. Confidence intervals (CI) for the binomial proportion of RVF and Brucellosis seropositivity were calculated using the Clopper-Pearson method, implemented via the “binomial” function of the “DescTools” package^81^. Time series data were visualised using ggplot2^82^ and the tidyverse packages^82^ to explore the temporal patterns in seropositivity.

To identify the factors associated with IgG seropositivity for RVF and brucellosis in a study population of 184 cattle, goats, and sheep, binary logistic regression analyses were conducted separately for each disease. The small number (3/184) of IgM-positive sera precluded their use as a dependent variable. Thus, IgG test status was used as the binary outcome (seropositive or seronegative) for both RVF and brucellosis models. Associations were quantified using adjusted odds ratios (ORs), where OR > 1 indicated increased odds of seropositivity, OR < 1 indicated decreased odds, and an OR = 1 suggested no association. The 95% CI for the ORs were calculated, with intervals excluding 1 indicating statistical significance (*p *< 0.05). Model fit was evaluated using the Hosmer–Lemeshow goodness-of-fit test (*p* > 0.05), indicating adequate fit^83^, and discrimination was assessed via the area under the receiver operating characteristic (ROC) curve.

In this study, the sensitivity analyses were performed to assess the robustness of the logistic regression results, given the relatively small sample size (n = 184) and the short data collection period (3 months). Specifically, we re-ran the multivariate models under alternative data and model specifications to verify that individual data points or modelling assumptions did not drive the associations observed. The following checks were performed: (1) Temporal sensitivity: models were re-fitted after excluding records from the earliest and latest sampling dates to confirm that results were not influenced by temporal clustering of abortion events. (2) Variable inclusion criteria: We varied the inclusion threshold for univariate screening (*p* < 0.15 vs *p* < 0.20) and found no change in the identity or direction of significant predictors. (3) Confounder removal: Each forced biological variable (e.g., stage of pregnancy, animal host) was removed in turn to ensure that model coefficients remained stable. Across all scenarios, adjusted odds ratios and their confidence intervals remained qualitatively similar, indicating that the models were robust to reasonable variations in data and modelling choices.

Figures 2,4, and all logistic regression analyses were performed using the glm function in R.

Mr Michael Wambi generated Fig. 1. First, administrative boundaries (district and sub-counties delineations), geographical features such as rivers, and boundaries of Lake Mburo National Park were obtained from the Uganda Bureau of Statistics (UBOS)–Uganda’s national statistics office^63^. All these datasets are open source and publicly available for use with a requirement for citation and acknowledgement upon use. These were downloaded as Uganda 2020 shapefile^84^ and were imported into the Geographic Information System (GIS) environment (ArcGIS)^85^ for processing. First, the data was processed to ensure all data layers were in a consistent geographic coordinate system, which allowed for spatial alignment. Then, the datasets were overlaid (administrative boundaries, rivers) to create a composite map (Fig. 1). For Fig. 3, it was generated by Dr Robert Ofwete, who used the timeliness of events data/field notes captured by the investigation team to create Fig. 2 in ArcGIS and Adobe Acrobat Reader–a PDF editor^86^.

## Supplementary Information

Below is the link to the electronic supplementary material.

Supplementary Material 1

## Data Availability

The data supporting this manuscript are all included in this manuscript. More information, including the statistical analysis that we did, is available (open access) at ( [https://github.com/abelwalekhwa/Livestock/_Abortions] ). For more information regarding this study, feel free to contact the corresponding author at [wabelwilson@gmail.com](mailto:wabelwilson@gmail.com).
