# Supplementary material for: Exploring community-based reporting of livestock abortions for rift valley fever and brucellosis surveillance in Uganda: a pilot study
Source: Sci Rep. 2025 Nov 28;15:42641. doi: 10.1038/s41598-025-26710-w (PMC12663114; doi:10.1038/s41598-025-26710-w)
Supplement: Supplementary file 1 — Supplementary Material 1 [file 41598_2025_26710_MOESM1_ESM.pdf]

Supplementary Information

S1 Table: Period for reporting of abortion cases

Variable	Before call center establishment<br>N = 10	Target period (within 14 days)<br>N = 174	Total
**Host**			
Goats	0	66	66
Cattle	10	96	106
Sheep	0	12	12
**Sub county**			
Bugango Town council	6	8	14
Endinzi	0	1	1
Endinzi Town council	0	1	1
Kakamba	0	17	17
Kashumba	0	40	40
Kikagati	0	6	6
Mbaare	4	10	14
Ngarama	0	13	13
Ruborogota	0	19	19
Rugaaga	0	43	43
Rushasha	0	12	12
Rwanjogyera	0	4	4
**Stage of pregnancy**			
Early stage (1-3 months)	4	69	73
Middle stage (4-6 months)	6	56	62
Late stage (7-9 months)	0	45	45
Not sure	0	4	4
**Environmental features**			
A	0	81	81
B	0	52	52
C	0	15	15
D	10	26	36
**History of animal movement**			
No	10	171	181
Yes	0	3	3
**Total**			**184**

S2; Logistic regression output for IgG Brucellosis Model

Variable	Estimate	Std. Error	z value	Pr(>|z|)	Null_deviance
(Intercept)	-18.747	3956.180	-0.005	0.996	190.085
host_brucellosisCattle	16.760	3956.180	0.004	0.996	190.085
host_brucellosisGoat	17.766	3956.180	0.004	0.996	190.085
host_brucellosisSheep	0.709	4165.296	0.000	0.999	190.085
environmental_featuresB	0.689	0.459	1.500	0.134	190.085
environmental_featuresC	0.288	0.700	0.412	0.680	190.085
environmental_featuresD	-0.560	0.646	-0.866	0.386	190.085
stage_pregnancyLate stage (7-9 months)	0.493	0.516	0.954	0.339	190.085
stage_pregnancyMiddle stage (4-6 months)	0.369	0.478	0.772	0.439	190.085
stage_pregnancyNot sure	-14.811	1842.029	-0.008	0.993	190.085
history_animal_movementYes	-14.948	2076.100	-0.007	0.994	190.085

S3 Appendix: Questionnare (Replicated from Predictive Factors and Risk Mapping for Rift Valley Fever Epidemics in Kenya [1]

Tool 1 (To be filled in by the call center)

Checklist for Community early warning system and reporting of abortions
Every entry is an abortion

1. Unique Id

2. Subcounty where abortion is reported

3. Village

4. Name of the reporter

5. Telephone number

6. Host species

- ◯ Cattle
- ◯ Goats
- ◯ Sheep

7. Age

8. Breed

- ◯ Local
- ◯ Exotic
- ◯ Mixed

9. Number of abortions reported

10. Action taken

- ◯ Ignored
- ◯ Investigated
- ◯ Referred

MAKERERE UNIVERSITY
SCHOOL OF PUBLIC HEALTH
APPROVED
VALID UNTIL
★ 02 FEB 2024 ★
RESEARCH & ETHICS COMMITTEE
P O BOX 7072, KAMPALA

11. Reporting platform

- ☐ SMS
- ☐ Facebook
- ☐ Whatsapp
- ☐ Calls

12. Date reported to the call center

yyyy-mm-dd

Tool 2 (To be filled in the community)

1. Unique Id

____

2. Gender of the respondent

- ◯ Male
- ◯ Female
- ◯ Prefer not to say

3. Age

____

4. Education level

- ◯ PhD
- ◯ Masters
- ◯ Bachelors
- ◯ Diploma
- ◯ Certificate
- ◯ Advanced level
- ◯ Ordinary level
- ◯ Illiterate

5. Occupation

____

6. Household size

____

7. Subcounty

- ◯ Birere
- ◯ Endizi
- ◯ Kabingo
- ◯ Kabuyanda
- ◯ Kashumba
- ◯ Kikagate
- ◯ Masha
- ◯ Nyakitunda
- ◯ Ngarama
- ◯ Rugaaga

8. Parish

____

9. Village

____

10. Date for the alert

yyyy-mm-dd

11. Date for investigation

yyyy-mm-dd

12. Date for vaginal swab collection

yyyy-mm-dd

13. Vaginal swab code

____

MAKERERE UNIVERSITY
SCHOOL OF PUBLIC HEALTH
APPROVED
VALID UNTIL
★ 02 FEB 2024 ★
RESEARCH & ETHICS COMMITTEE
P O. BOX 7072, KAMPALA

14. Any clinical presentation of the animal

- [ ] High mortality of new-born
- [ ] Sudden onset of abortion among pregnant animals
- [ ] Weakness /Unsteady gait
- [ ] Mucopurulent nasal discharge
- [ ] Profuse fetid diarrhea
- [ ] High fever
- [ ] I don't know

15. Is the animal vaccinated

- ◯ Yes
- ◯ No

16. Herd size

____

17. Number of animals vaccinated for RVF

____

18. Any environmental features observed

- [ ] Bushes
- [ ] Recent rainfall (less than 14 days)
- [ ] Stagnant water
- [ ] Shrubs
- [ ] Heavy forests
- [ ] Others

Others

____

19. History of animal movement

- ◯ Yes
- ◯ No

If yes state;

____

20. Origin

____

21. Destination

____

22. Image of animal

Click here to upload file. (< 10MB)

23. GPS coordinates

latitude (x.y °)

____

longitude (x.y °)

____

altitude (m)

____

accuracy (m)

____

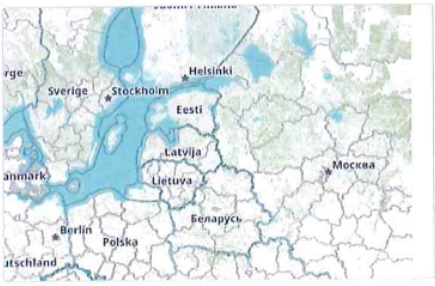

MAKERERE UNIVERSITY
SCHOOL OF PUBLIC HEALTH
APPROVED
VALID UNTIL
02 FEB 2024
RESEARCH & ETHICS COMMITTEE
P O BOX 7072, KAMPALA

Tool 3 (To be filled in by the Lab Assistant)

1. Unique Id

____

2. Vaginal swab id

____

3. Number of tests done

____

4. Results of ELISA, IgM

____

5. Mean OD value of each serum sample

____

6. Results of ELISA, IgG

____

7. Mean OD value of each serum sample

____

8. Id of the lab technician

____

9. Date for the results

yyyy-mm-dd

MAKERERE UNIVERSITY
SCHOOL OF PUBLIC HEALTH
APPROVED
VALID UNTIL
02 FEB 2024
RESEARCH & ETHICS COMMITTEE
P O. BOX 7072, KAMPALA

[1]Munyua PM, Murithi RM, Ithondeka P, Hightower A, Thumbi SM, Anyangu SA, Kiplimo J, Bett B, Vrieling A, Breiman RF *et al*: **Predictive Factors and Risk Mapping for Rift Valley Fever Epidemics in Kenya**. *PLoS One* 2016, **11**(1):e0144570.

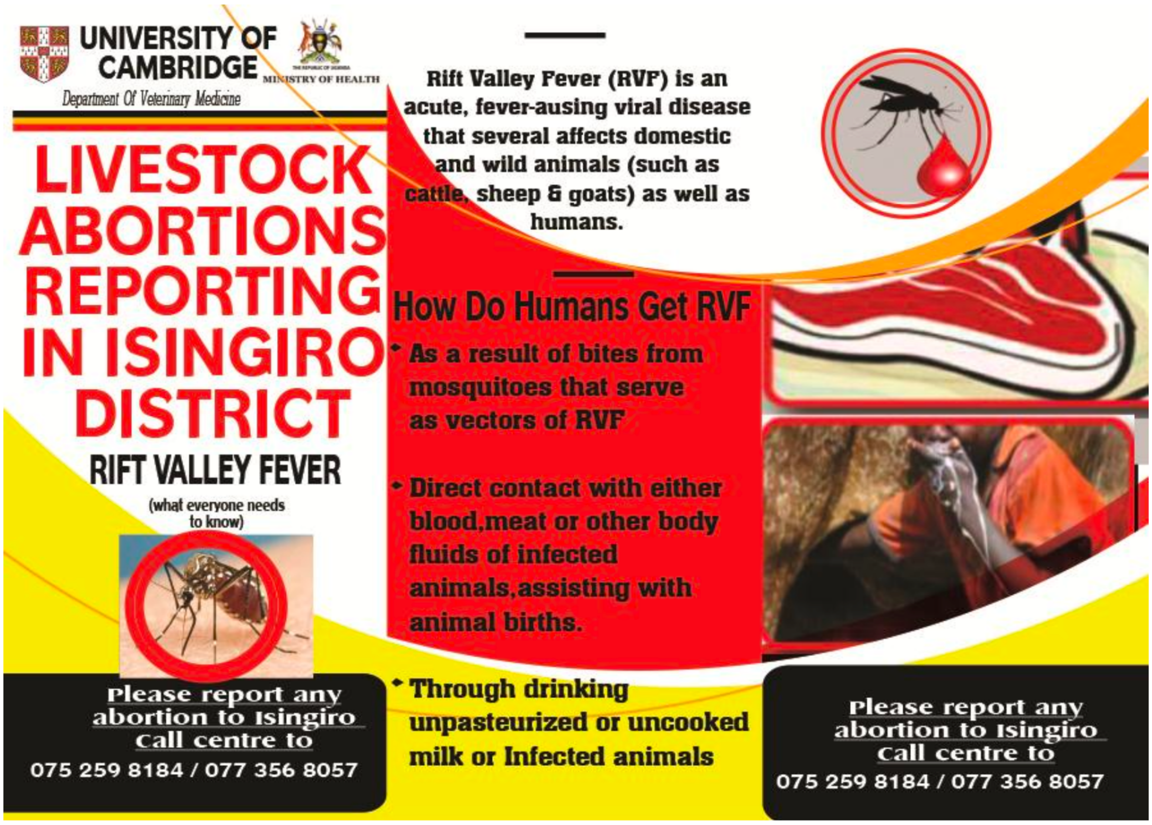

Figure 1: IEC Materials for RVF used during sensitizations (Front page)

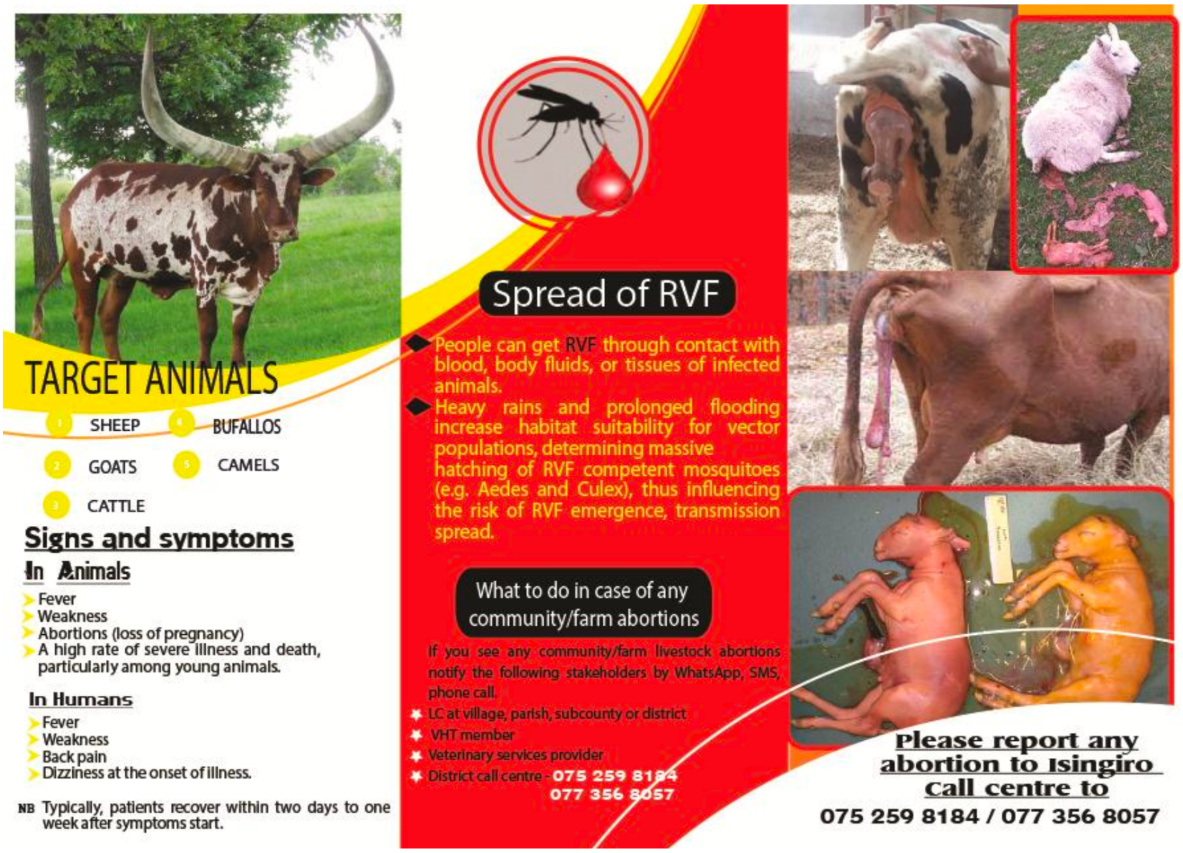

S3 Appendix: IEC Materials for RVF used during sensitizations (Back page)
